# Conversion of Rutin, a Prevalent Dietary Flavonol, by the Human Gut Microbiota

**DOI:** 10.3389/fmicb.2020.585428

**Published:** 2020-12-21

**Authors:** Alessandra Riva, Ditta Kolimár, Andreas Spittler, Lukas Wisgrill, Craig W. Herbold, László Abrankó, David Berry

**Affiliations:** ^1^Centre for Microbiology and Environmental Systems Science, Department of Microbiology and Ecosystem Science, Division of Microbial Ecology, University of Vienna, Vienna, Austria; ^2^Faculty of Food Science, Department of Applied Chemistry, Szent István University, Budapest, Hungary; ^3^Core Facility Flow Cytometry, Department of Surgery, Research Lab, Medical University of Vienna, Vienna, Austria; ^4^Division of Neonatology, Pediatric Intensive Care and Neuropediatrics, Department of Pediatrics and Adolescent Medicine, Medical University of Vienna, Vienna, Austria; ^5^Joint Microbiome Facility of the Medical University of Vienna and the University of Vienna, Vienna, Austria

**Keywords:** dietary bioactives, rutin, gut microbiota, fluorescence activated cell sorting, rutin metabolism, inter-individual variability

## Abstract

The gut microbiota plays a pivotal role in the conversion of dietary flavonoids, which can affect their bioavailability and bioactivity and thereby their health-promoting properties. The ability of flavonoids to metabolically-activate the microbiota has, however, not been systematically evaluated. In the present study, we used a fluorescence-based single-cell activity measure [biorthogonal non-canonical ammino acid-tagging (BONCAT)] combined with fluorescence activated cell sorting (FACS) to determine which microorganisms are metabolically-active after amendment of the flavonoid rutin. We performed anaerobic incubations of human fecal microbiota amended with rutin and in the presence of the cellular activity marker L-azidohomoalanine (AHA) to detect metabolically-active cells. We found that 7.3% of cells in the gut microbiota were active after a 6 h incubation and 26.9% after 24 h. We then sorted BONCAT-positive cells and observed an enrichment of *Lachnospiraceae* (*Lachnoclostridium* and *Eisenbergiella*), *Enterobacteriaceae, Tannerellaceae*, and *Erysipelotrichaceae* species in the rutin-responsive fraction of the microbiota. There was marked inter-individual variability in the appearance of rutin conversion products after incubation with rutin. Consistent with this, there was substantial variability in the abundance of rutin-responsive microbiota among different individuals. Specifically, we observed that *Enterobacteriaceae* were associated with conversion of rutin into quercetin-3-glucoside (Q-glc) and *Lachnospiraceae* were associated with quercetin (Q) production. This suggests that individual microbiotas differ in their ability to metabolize rutin and utilize different conversion pathways.

## Introduction

Flavonoids are a group of bioactive polyphenolic compounds present in a wide variety of plant-based foodstuffs. Rutin (Q-3-*O*-rutinoside) is a flavonol glycoside composed of Q and rutinose, a disaccharide of rhamnose and glucose, and represents the most consumed flavonol (3.75% in Europe and in the United Kingdom) (Zamora-Ros et al., 2016). Dietary sources of rutin include tea, green asparagus, onions, buckwheat, wine, eucalyptus, apples, as well as berries (de Araujo et al., 2013; Kumar and Pandey, 2013; Amaretti et al., 2015). Rutin has been shown to have anti-oxidant properties (Ghorbani, 2017) and to exert anti-aging effects on human dermal fibroblasts and human skin (Choi et al., 2016). It also has anti-neurodegenerative properties (Enogieru et al., 2018) and exhibits protective effects against hyperglycemia, dyslipidemia, liver damage, and cardiovascular disorders (Ghorbani, 2017). Additionally, the rutin degradation products Q-glc and Q have been found to have anti-inflammatory, anti-oxidant, and anti-mutagenic properties (Gibellini et al., 2011; Kumar and Pandey, 2013; Hobbs et al., 2018). Q-glc also possesses anti-hypertensive, hypolipidemic effects (Gibellini et al., 2011; Kumar and Pandey, 2013; Hobbs et al., 2018) and Q has been reported to ameliorate atherosclerosis and dyslipidemia (Salvamani et al., 2014).

The bioavailability of these dietary flavonoids depends on intestinal absorption, which is determined by their chemical composition and, in particular, by the nature of glycosylation (Matsumoto et al., 2004). The glyco-conjugates of Q are poorly absorbed in the upper intestinal tract and accumulate in the large intestine. In the colon, members of the gut microbiota can hydrolyze rutin or other glyco-conjugates, removing the sugar moiety and permitting the absorption of the aglycone (Cardona et al., 2013; Amaretti et al., 2015). Therefore, the colonic microbiota is responsible for the extensive breakdown of the original flavonoid structures into low-molecular-weight phenolic metabolites (Cardona et al., 2013). Currently, it is estimated that 500–1000 different microbial species inhabit the gastrointestinal tract, reaching the highest concentration in the colon (up to 10^12^ cells per gram of feces) (Thursby and Juge, 2017). Bacteria that metabolize rutin possess α-rhamnosidases that transform rutin into Q-glc and/or β-glucosidases that either convert Q-glc into Q (Braune and Blaut, 2016) or convert rutin directly into Q (Olthof et al., 2003). A limited number of bacteria have so-far been shown to have rutin-metabolizing capabilities in pure culture. α-rhamnosidases involved in deglycosylation of flavonoids have been characterized in *Lactobacillus acidophilus*, *Lactobacillus plantarum* (Beekwilder et al., 2009), and *Bifidobacterium dentium* (Bang et al., 2015). The capability to degrade rutin into Q was reported for *Bacteroides uniformis*, *Bacteroides ovatus* (Bokkenheuser et al., 1987), and *Enterococcus avium* (Shin et al., 2016). *Parabacteroides distasonis* was shown produce both Q-glc and Q via α-rhamnosidase and β-glucosidase activity (Bokkenheuser et al., 1987), and *Eubacterium ramulus* and *Enterococcus casseliflavus* are able to convert Q-glc in Q (Schneider et al., 1999).

Previous studies of rutin conversion by gut bacteria have involved screening strain collections, which gives limited insight into identifying which bacteria are actually involved in metabolizing rutin in the complex gut microbial community. In the present study, we identified cells in the gut microbiota that became metabolically-active upon rutin amendment using the cellular activity marker L-azidohomoalanine (AHA). By sorting active cells and profiling active and total communities using 16S rRNA gene amplicon sequencing, we were able to identify specific taxa enriched in rutin-treated samples. We observed marked inter-individual variability in both the extent of rutin degradation product formation as well as the abundance of the rutin-responsive microbial community. Our findings present new insights into rutin metabolism by different microbiotas in healthy individuals, which will be useful for future studies on flavonol metabolism in health as well as in disease conditions.

## Materials and Methods

### Sample Collection

Fresh fecal samples were collected from 10 healthy subjects (seven females and three males, age mean ± SD:30.5 ± 5.8; BMI:mean ± SD: 22.19 ± 2.9). All participants followed an omnivore diet. Participants with antibiotic, probiotic, or prebiotic usage in the previous 6 months were excluded. The study was approved by, and conducted in accordance, with the University of Vienna ethics committee (Reference number 00161) and written informed consent was signed by all enrolled participants.

### Anaerobic Incubations

Fresh stool samples were immediately introduced into an anaerobic tent (85% N_2_, 10% CO_2_, 5% H_2_). All reagents were introduced into the anaerobic tent 2 days before the experiment to ensure that they were anaerobic by the start of the experiment. 10 mL phosphate-buffered saline (PBS) was added to 1 g of fecal sample. The suspension was homogenized by vigorous shaking and vortexing. Samples were left for 15 min to allow large particles to settle and subsequentally serially-diluted 1:10 twice. The serial 1:10 dilutions were necessary to avoid background noise and autofluorescence in the Cy5 [biorthogonal non-canonical ammino acid-tagging (BONCAT)] signal. Samples were incubated in autoclaved Hungate tubes in the presence of 1 mM of the non-canonical amino acid *L*-AHA (baseclick GmbH, Germany) and 500 μM rutin dissolved in dimethyl sulfoxide (DMSO; Sigma–Aldrich). A negative control containing DMSO and a positive control with 2 mg/mL of glucose were used for each experiment. An abiotic control for each time point was included to assess the chemical stability of rutin under the incubation conditions. Samples were incubated under anaerobic condition with a final volume of 5 mL for 0, 6, or 24 h. Subsequently, samples were centrifuged at 14,000 r/min for 10 min and the supernatant was collected and diluted with equal volume of pure acetonitrile (ACN) in order to stabilize the supernatant samples and then stored at −20°C for liquid chromatography-high resolution mass spectrometry (LC-HRMS) analysis. Part of the sample was frozen for nucleic acid extraction and part was washed twice in PBS and then fixed in 1:1 ethanol:PBS for FACS sorting.

### Liquid Chromatography-High Resolution Mass Spectrometry (LC-HRMS)

Acetonitrile (LC-MS grade) and formic acid were purchased from VWR. High purity water (18.2 MΩ cm^–1^) was used for dilution of samples and the preparation of mobile phases (Milli-Q Synergy/Elix water purification system, Merck). Authentic reference standards of rutin, Q-3-*O*-glucoside and Q were purchased from Sigma–Aldrich (>94%, HPLC) and Extrasynthese (>99%, HPLC), respectively. Supernatants from the incubations were immediately centrifuged and aliquots for HPLC analysis were removed and equal volume of ACN was added. Samples were kept frozen until analysis. The ACN-stabilized samples were thawed and homogenized by vortexing. 100 μL was diluted 1:4 in water to decrease ACN content to 10%. The diluted sample was filtered through a 0.22 μm pore polytetrafluoroethylene syringe filter (Cronus, LabHut Ltd.), and 5 μL were injected into the LC system. Chromatographic separation was achieved on a Phenomenex Kinetex EVO C18 100 × 2.1 mm, 2.6 μm column utilizing an Agilent 1200 HPLC system. The column was operated at 30°C. The binary mobile phase consisted of H_2_O with 0.1% formic acid (eluent A) and ACN (eluent B). The flow rate was set to 0.4 mL min^–1^. Gradient separation was started at 5% B and linearly increased to reach 90% in 9 min. The eluent was kept constant at 90% B until 11.5 min and then the column was re-equilibrated at the initial conditions for 11.5 min. The effluent of the LC system was connected to an Agilent 6530 high-resolution, accurate mass quadrupole/time-of-flight mass spectrometer equipped with a dual sprayer electrospray ion source (ESI-Q/TOFMS). The mass spectrometry was run in full scan (MS-only) mode scanning from *m/z* 50–1700 in negative ionization mode. A continuous reference mass correction was applied using purine and HP-921 [Hexakis(1H,1H,3H-perfluoropropoxy)phosphazene] as reference substances. The ion source temperature was maintained at 325°C and capillary and fragmentor voltages were set to −4000 and 140 V, respectively. The Mass Hunter Workstation software package (B02.01) was used for data acquisition and data evaluation. Quantification of rutin, Q glucoside, and Q was performed with external calibration using reference standards.

### BONCAT Labeling of Microbial Cells

Cu(I)-catalyzed click labeling of chemically-fixed microbial cells was performed on slides as described previously (Hatzenpichler et al., 2014). Briefly, fixed samples were immobilized on glass slides, dried in a 46°C hybridization oven, and dehydrated and permeabilized by placing slides for 3 min sequentially in 50, 80, and 96% ethanol. Then, 1.25 μL of 20 mM CuSO4, 2.50 μL of 50 mM tris[(1-hydroxypropyl-1H-1,2,3-triazol-4-yl)methyl]amine (THPTA) (baseclick GmbH, Germany), and 0.30 μL of alkyne dye (in DMSO) (Jena Bioscience, Germany) were mixed and allowed to react for 3 min at room temperature (RT) in the dark. In the meantime, 12.5 μL of freshly-prepared 100 mM sodium ascorbate (Sigma–Aldrich) and 12.5 μL of 100 mM aminoguanidine hydrochloride (Sigma–Aldrich) were added to 221 μL 1 × PBS (pH 7.4). Then, the dye premix was added to this solution, the tube inverted once and samples were covered by 30 μL of solution. Slides were transferred into a humid chamber and incubated in the dark at RT for 30 min. Afterward, slides were washed three times for 3 min each in 1 × PBS and then treated with an increasing ethanol series (3 min each in 50, 80, and 96% ethanol) and air-dried (Hatzenpichler et al., 2014). 1:1000 DNA stain, 4′, 6 diamidino-2-phenylindole (DAPI) solution (in PBS), was applied for 5 min and then slides were washed in cold MILLI-Q water (Millipore GmbH, Vienna, Austria). Samples were embedded with CitiFluor (Agar Scientific Ltd., Stansted, United Kingdom) if used immediately or stored at −20°C. Representative BONCAT pictures of a fecal sample incubated with rutin at 6 and 24 h and a negative control containing DMSO are shown in Supplementary Figure 1. Representative BONCAT picture of a fecal sample incubated with the glucose positive control is shown in Supplementary Figure 2. For FACS sorting, in-solution click labeling was performed immediately before FACS sorting. For click labeling, 300–500 μL fixed samples were centrifuged at 10,000 r/min for 10 min and re-suspended in 96% ethanol. The pellet was left for 3 min at RT and then centrifuged 10,000 r/min for 5 min. A master mix containing the dye solution was prepared as described above. Samples were suspended in 60–100 μL of solution and incubated in the dark at RT for 30 min. Afterward, samples were washed three times by centrifugation with 1x PBS (Hatzenpichler et al., 2014). Immediately before sorting, samples were filtered with a 35 μm nylon mesh using BD tubes 12 × 75 mm (BD, Germany).

### Image Acquisition and Analysis

20–30 images were collected for each sample with an epifluoresce microscope (Zeiss-Axio-imager, Germany). Image analysis was performed using the software *digital image analysis in microbial ecology* (Daime) and the biovolume fraction, the fraction of BONCAT-labeled biomass (Cy5-labeled) relative to the total biomass (DAPI-labeled), was calculated (Daims et al., 2006).

### Fluorescence Activated Cell Sorting (FACS)

For flow cytometry sorting, bacteria were labeled in Cy5 dye as previously described, and analyzed on an ultra high-speed cell sorter MoFlo Astrios EQ (Beckman Coulter, Brea, CA, United States) using the Summit v6.2 software (Beckman Coulter). To standardize the daily measurement and to assess the size of the bacteria, calibration beads (silica beads 100, 500, and 1000 nm, Kisker Biotech, Steinfurt, Germany) having a refractive index close to biological material were recorded. The sorting of Cy5-labeled bacteria was performed as follows: In a first scatter plot, the 561 nm SSC Height-Log parameter was set vs. the 488 nm FSC1 Height-Log parameter. To reduce electronic noise, the triggering signal was set on the 561 nm SSC parameter. A second dot plot 488 nm FSC1-Height-Log vs. 488 nm SSC-Height-Log showed in a first measurement the different sizes of the silica beads and in the following measurements the scattering of the bacteria. Bacteria were then pre-gated and displayed on a third scatter plot with 488 nm SSC area log axes vs. 640 nm 671/30-Area-Log axes. Cy5-positive bacteria were then sorted out into tubes with a maximum event rate of 50,000 events per second. Reanalysis of the samples showed a purity of >99%. An overview of the gating strategy and FACS selectivity analysis is shown in Supplementary Figures 3, 4. In order to assess the technical reproducibility of the method, absolute cell count was performed with the cell sorter FACS Melody (BD, Germany) and BONCAT-positive cells were counted in triplicate for each sample (Supplementary Figures 5A,B). Absolute counting beads (CountBright^TM^, Invitrogen, ThermoFisher Scientific, Austria) were used for cell counts according to the manufacturer’s instructions.

### DNA Extraction and 16S rRNA Gene Amplicon Sequencing

DNA extraction was performed for both total microbial community and the FACS-sorted fraction using the QiAmp mini DNA extraction kit (Qiagen) following the protocols for bacteria according to the manufacturer’s instructions. PCR amplification was performed with a two-step barcoding approach according to Herbold et al. (2015) using 16S rRNA gene primers targeting most bacteria (V3–V4 region of the 16S rRNA gene) (S-D-Bact-0341-b-S-17 [5′-CCTACGGGNGGCWGCAG-3′] and S-D-Bact-0785-a-A-21 [5′-GACTACHVGGGTATCTAATCC-3′]. The barcoded amplicons were purified with ZR-96 DNA Clean-up Kit (Zymo Research, United States) and quantified using the Quant-iT^TM^ PicoGreen^®^ dsDNA Assay (Invitrogen, United States) (Herbold et al., 2015). An equimolar library was constructed by pooling samples, and the resulting library was sent for sequencing on the Illumina MiSeq platform at Microsynth AG (Balgach, Switzerland).

### Sequence Processing and Data Analysis

16S rRNA gene sequence data were sorted into libraries according to Herbold et al. (2015) and processed into amplicon sequence variants (ASVs) using the Divisive Amplicon Denoising Algorithm (DADA2) (Callahan et al., 2016) and classified using the RDP classifier (Wang et al., 2007) as implemented in Mothur (Schloss et al., 2009). Sequencing libraries were subsampled to a smaller number of reads than the smallest library (subsampled to 1000 reads) to avoid biases related to uneven library depth. 1000 reads were sufficient to maintain a high coverage per library (mean Good’s coverage: 99%). 16S rRNA gene sequence data have been deposited in the NCBI Short Read Archive under PRJNA622517. Statistical analysis was performed using R statistical software^1^. Statistical analysis to compare samples groups was performed using ANOVA, and with the R package DEseq2 (Love et al., 2014). The statistical significance of factors affecting microbiota composition was evaluated using non-parametric permutational multivariate analysis of variance (perMANOVA), significant clustering of groups was evaluated with analysis of similarities (ANOSIM), ordination was performed using redundancy analysis (RDA) and non-metric multidimensional scaling (NMDS) in the vegan package in R (Oksanen et al., 2010). Relative standard deviation (RSD) was calculated as standard deviation (SD)/mean to determine inter-individual variability across participants. Microbial community variation within participant was further determined using the UpSetR package in R, which employs a matrix-based layout to show intersections of sets and their sizes (Conway et al., 2017). Alpha and beta diversity metrics were also calculated with the vegan package. Variables are expressed as mean ± SD. A probability value (*p*-value) less than 0.05 was considered statistically significant and *p*-values were adjusted with the false discovery rate (FDR) method in the case of multiple comparison. Statistical analysis to compare producer groups was performed using ANOVA and Tukey test for multiple comparisons.

### Screening Bacterial Genomes for Alpha-Rhamnosidase Genes

Uniprot^2^ was queried using “alpha-L-rhamnosidase” as a query and filtered for “reviewed” entries. Entries were manually examined for confirmation of activity and to compile pfam motifs (El-Gebali et al., 2019). A single pfam motif, Bac_rhamnosid6H (PF17389), was common to all genes with confirmed alpha-rhamnosidase activity. Uniprot was subsequently queried using PF17389 and filtered for “reviewed” entries to determine specificity. All reviewed entries for PF17389 possessed confirmed alpha-rhamnosidase activity. All publicly-available genomes available at NCBI as 24 September 2020 (*N* = 268,330) were screened using PF17389 with hmmsearch (hmmer3^3^) and genes matching the pfam (E < 10^–6^ and alignment over at least 300 amino acids) were extracted from the genomes. All extracted genes were back-screened against the Pfam-A database with hmmscan (hmmer3) and only those genes that matched PF17389 as a best-hit were retained as putative alpha-rhamnosidase. Genus-level assignments were inferred by string-matching genus labels (e.g., *Escherichia*) to the organism name associated to each searched genome.

## Results

### Biotransformation of Rutin by the Gut Microbiota

**FIGURE 1:**
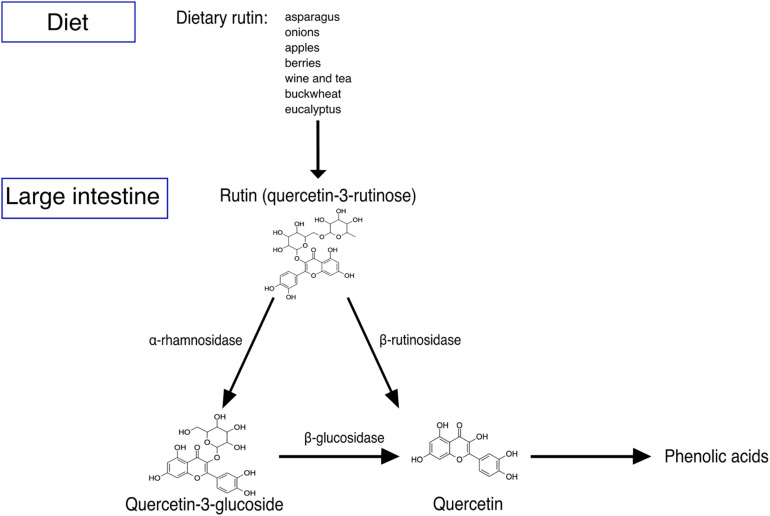
Schematic representation of rutin degradation. Rutin is present in a wide variety of foods. Rutin is not well absorbed in the small intestine of humans, and thus is transported into the colon and metabolized by the gut microbiota into quercetin-3-glucoside and then quercetin or directly into quercetin. Quercetin may be subsequently degraded mainly into different phenolic acids.

**FIGURE 2:**
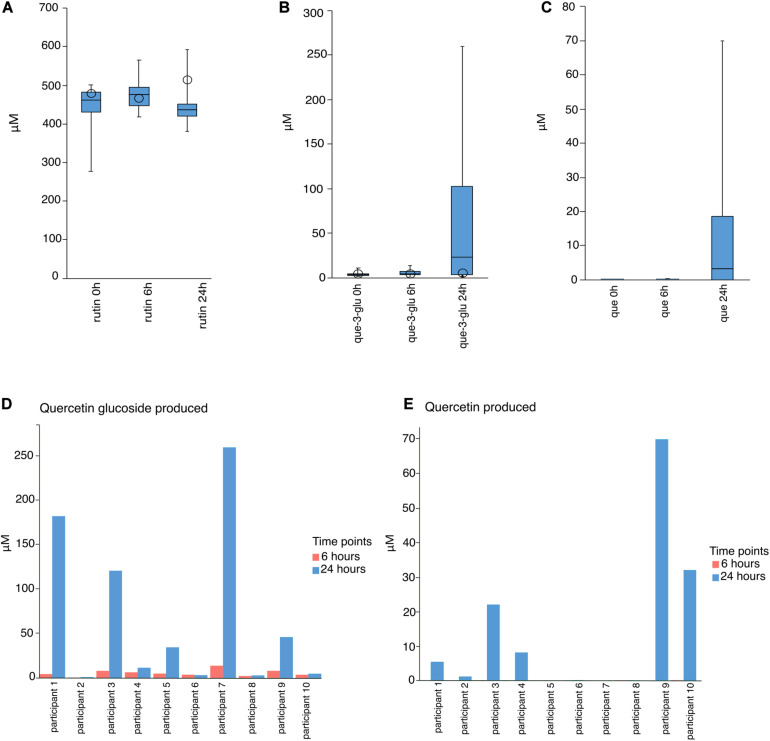
Molar concentrations of rutin, quercetin-3-glucoside (Q-glc), and quercetin (Q) after 0, 6, and 24 h incubation. **(A–C)** Box and whisker plots of the combined results of incubation of rutin with the gut microbiota of 10 participants, as obtained from LC-HRMS analysis. Open circles indicate results of abiotic controls. **(D)** Q-glc and **(E)** Q concentrations in each incubation after 6 and 24 h.

In order to characterize the biotransformation of rutin by the gut microbiota and to identify metabolically-active bacteria after rutin amendment, we performed anaerobic incubations of freshly-collected stool contents amended with 500 μM rutin (Figure 1). This concentration is in line with previous studies and is consistent with a reasonable dietary intake (Amaretti et al., 2015; Zamora-Ros et al., 2016). Over the course of the incubations, there was a slight but not statistically-significant reduction in rutin (one-way ANOVA, *p* = 0.41, n = 10, molar recovery: 97%; Figure 2A), probably caused by the high dilution applied in the anaerobic incubation. However, Q-glc and Q appeared after incubation with biomass (*p* = 0.019 and 0.036 for Q-glc and Q, respectively, *n* = 10; Figures 2B,C), indicating that rutin was actively transformed by the gut microbiota. Low levels of Q-glc were present in all time zero samples (Figure 2B), which may be because rutin preparations were either not completely purified or chemically degraded to Q-glc during storage. Interestingly, there was substantial variation in the amount of Q-glc and Q formed during incubation with the gut microbiota, suggesting considerable inter-individual variability in the capacity to metabolize rutin by different gut microbial communities (Figures 2D,E). Moreover, we have screened the samples for some known Q metabolites (dihydroxyphenyl propionic acid hydroxyphenylacetic acid, dihydroxyphenylacetic acid, dihydroxybenzoic acid, hydroxybenzoic acid) and only a few samples showed hits and only with minor signals. Therefore, these hits and suspected metabolites were not included in the data evaluation.

### The Core Metabolically-Active Microbiota in Rutin Amendments

**FIGURE 3:**
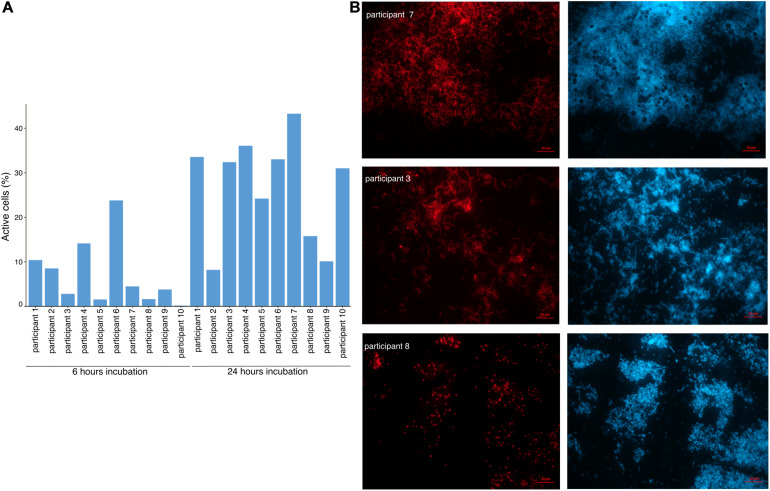
The rutin-activated microbiota. **(A)** Relative abundance of metabolically-active cells in incubations. Relative standard deviation (RSD) was 1.0 for time 6 h and 0.43 for time 24 h. **(B)** Representative microscopic images of samples from three participants showing variability in cell morphology between donors. Active cells are represented in red (BONCAT-Cy5) and all cells are stained in blue (DAPI).

**FIGURE 4:**
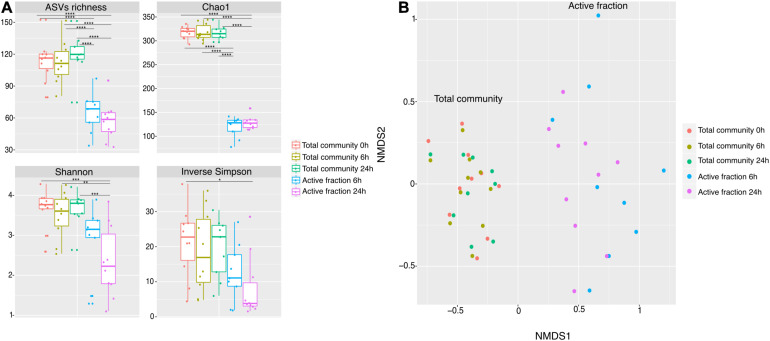
Microbiota richness and diversity in metabolically-active community in rutin incubations. **(A)** Observed ASVs, Chao1 estimated richness, Shannon diversity, and inverse Simpson diversity estimators show significant difference between time points, active fraction and total community [ASVs richness, Chao1 and Shannon (ANOVA: *p* < 0.0001, *n* = 50), Inv. Simpson (ANOVA: *p* = 0.020, *n* = 50)]. Multiple comparisons are represented in the figure as asterisks. **(B)** NMDS ordination shows samples separation between the total community and the active fraction.

**FIGURE 5:**
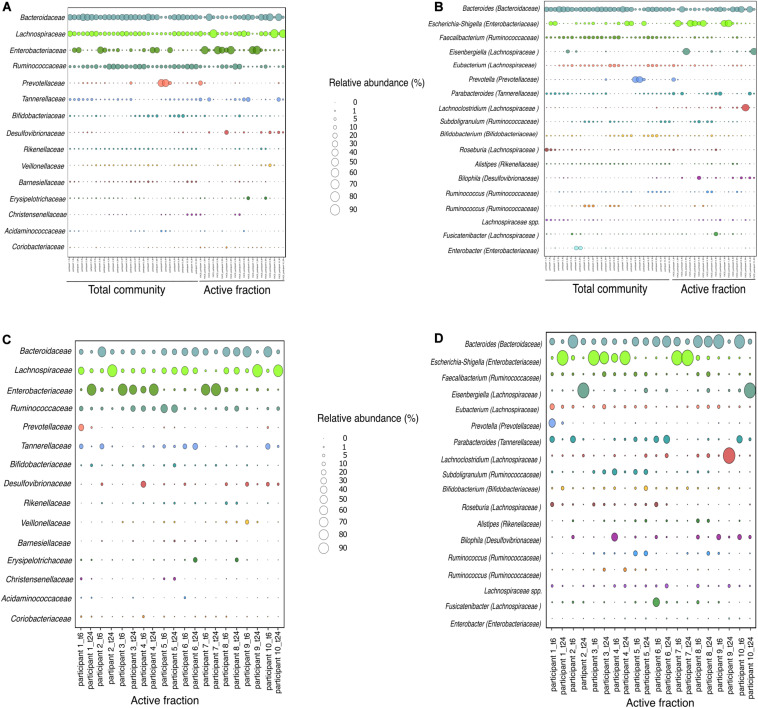
Relative abundance of bacterial taxa based on 16S rRNA gene amplicon sequencing at family and genus level for each participant. **(A,B)** Relative abundance of the total community and the active fraction at time 6 and 24 h. **(C,D)** Relative abundance of the active fraction at time 6 or 24 h. Family and genera with relative abundance > 0.5 and > 1%, respectively, is shown.

As the capacity of dietary polyphenols to modulate the activity and/or composition of the gut microbiota is still poorly understood, we aimed to identify the metabolically-active microbial taxa upon rutin amendment. Rutin amendment activated a subset of the microbiota, and active cells were detected in almost all samples after 6 h of incubation with rutin, with an increasing number after 24 h (6 h: 7.3 ± 7.3%, 24 h: 26.9 ± 11.7% [mean ± SD]; *t*-test: *p* = 0.0003, *n* = 20) (Figures 3A,B). The diversity and the composition of the total microbial community did not change significantly during the short-term incubations (Figures 4A,B; perMANOVA, *p* = 0.99), indicating that the applied incubation conditions did not appreciably modify the composition of the microbiota (i.e., there was no strong “bottle effect”). However, the rutin-active fraction of the community, as determined by BONCAT activity labeling and FACS sorting, was significantly different from the total community. The diversity of the active fraction was lower than the total community (ANOVA, *p* < 0.0001 for the alpha diversity metrics; Figure 4A), and the NMDS ordination showed a clear separation of samples between the total community and the active fraction (perMANOVA, *p* < 0.0001; ANOSIM, *p* = 0.001) (Figure 4B). The most abundant taxa detected in the active fraction were *Bacteroidaceae*, *Enterobacteriaceae*, *Lachnospiraceae*, *Tannerellaceae*, and *Ruminococcaceae* (Figure 5). We found that members of the *Enterobacteriaceae* (*Escherichia/Shigella*), *Tannerellaceae* (*Parabacteroides*), *Erysipelotrichaceae* (*Erysipelatoclostridium*), and *Lachnospiraceae* (*Lachnoclostridium* and *Eisenbergiella*) were significantly increased in the active fraction compared to their respective total community samples (negative binomial distribution, Wald test, *p* < 0.05, *n* = 20). We then determined the prevalence of putative alpha-rhamnosidases (Bac_rhamnosid6H [PF17389]) in publicly-available genomes belonging to genera that comprised the core rutin-active community.

We further determined the proportion of database genomes that encoded a-Rhamnosidase for rutin-active genera (Supplementary Figure 6A) and non-active genera (Supplementary Figure 6B) and, in agreement with our experimental results, we found that putative alpha-rhamnosidases are common in genomes belonging genera that responded to the rutin amendment. We then used a non-parametric test to show that genomes randomly drawn from rutin-active genera would be expected to contain an a-Rhamnosidase more often than genomes randomly drawn from rutin-non-active genera (Mann–Whitney one-tailed test, *p* = 0.041, Supplementary Figure 6C), suggesting that the active genera are more likely to have database representatives that contain a-Rhamnosidase.

### Microbial Community Composition Is Associated With Rutin Transformation Patterns

**FIGURE 6:**
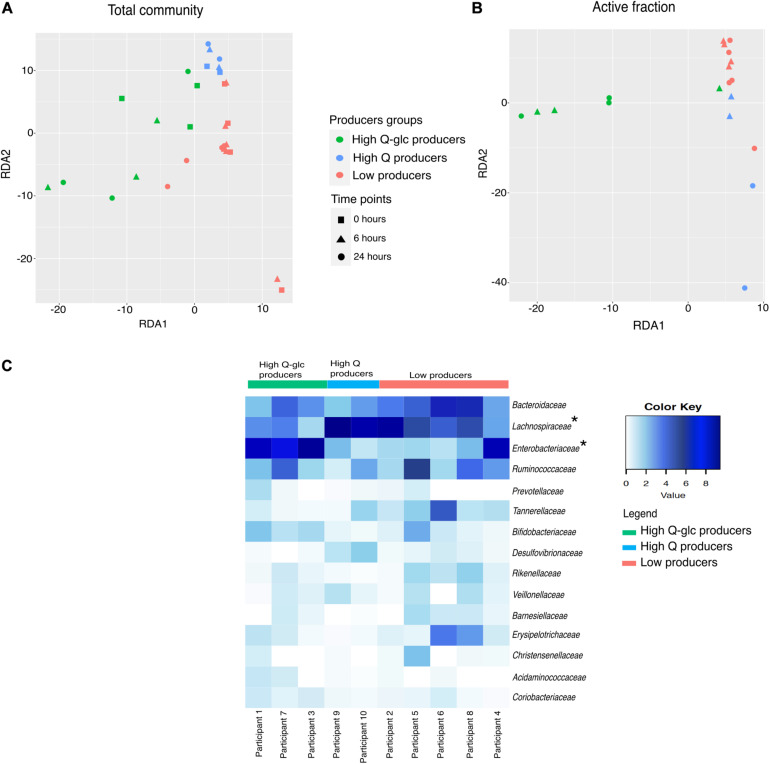
Samples clusters based on rutin degradation pattern. Redundancy analysis shows sample clustering by degradation pattern in both the **(A)** total community [constrained variance explained:18.6%, RDA1 (14%), RDA2 (4.8%)] and **(B)** active fraction [constrained variance explained: 40.5%, RDA1 (36.3%), RDA2 (4.1%)]. **(C)** Family-level heatmap showing the square root transformed relative abundance of the active fraction divided by producer groups at 24 h. Significant changes in relative abundance (Low producers vs. High producers) are indicated with asterisks. *Enterobacteriaceae* increased in relative abundance in the High Q-glc producers. (High Q-glc producers vs. Low producers, *p* = 0.0055, High Q-glc producers vs. High-Q producers, *p* = 0.0061). *Lachnospiraceae* increased in relative abundance in the high-Q producers (High-Q producers vs. Low producers, *p* = 0.0043, High-Q producers vs. High Q-glc producers, *p* < 0.00001).

We observed that there was a large variability in the ASVs that were enriched in the active fraction of the community across the incubations from different donors. Only 13/97 and 23/91 ASVs were shared among all incubations at 6 and 24 h, respectively (Supplementary Figures 7A,B). We confirmed that the technical variability associated with the BONCAT procedure was much smaller than the observed biological variability (ANOVA; technical variation: *r*^2^ = 0.00085, *p* = 0.878; biological variation: *r*^2^ = 0.999, *p* < 0.0001; Supplementary Figures 5A,B). We compared the inter-individual variability in the fraction of cells metabolically-activated by rutin with glucose, a compound that can be fermented by a large fraction of the gut microbiota, and found that inter-individual variability was higher in rutin amendments than glucose amendments (RSD 24 h rutin: 0.43, RSD 24 h glucose: 0.22; Figure 3A and Supplementary Figure 2). Consistent with this, we found that the microbial community varied significantly within participant in both the total community and active fraction (perMANOVA: *p* < 0.0001, Supplementary Figures 7C,D), which was in line with the observation that different cell morphologies were observed in the active fraction from different donors (Figure 3B). Though stool incubations from all participants led to the production of rutin degradation products, the pattern of rutin product formation was markedly different among different participants. We therefore divided the samples into the following groups: “High Q-glc producers,” “High Q producers,” and “Low producers.” Based on these categories, we observed a significant clustering of both total community and active fraction samples in the RDA ordination (perMANOVA, total community: *p* = 0.001, active fraction: *p* = 0.001) (Figures 6A,B). In the active fraction, we observed an enrichment of *Enterobacteriaceae* (*Escherichia-Shigella*) in High Q-glc producers, and this taxon represented the majority of sequences recovered in the active fraction by 24 h (ANOVA: 0.0002, *n* = 50). In contrast, High Q producers had an enrichment of *Lachnospiraceae* (*Lachnoclostridium* and *Eisenbergiella*), which was the dominant taxon in the active fraction by 24 h (ANOVA: 0.0257, *n* = 50). Low producers had a trend, though not statistically significant, toward enrichment in *Tannerellaceae* (*Parabacteroides*) and *Erysipelotricaceae* (*Erysipelatoclostridium*) in the active fraction (Figure 6C and Supplementary Figures 6, 8).

## Discussion

The gut microbiota plays a key role in the conversion of dietary flavonoids. Though conversion of flavonoids by the microbiota has gained increasing interest (Braune and Blaut, 2016), key microbial players in flavonoid metabolism remain poorly characterized (Cardona et al., 2013). In this study, we investigated metabolism of the flavonoid rutin by the human gut microbiota. Rutin conversion products were detected in all tested donor stool incubations, but there was a dramatic variation in the amount of Q-glc and Q produced by different microbiotas. This suggests inter-individual variability in preference or capability for rutin metabolism. This is in line with a previous report of high person-to-person variation in the concentration of phenolic acids between 2 and 24 h incubation of stool with rutin (Jaganath et al., 2009).

Alpha diversity was lower in the active fraction compared to the total community, implying that a subset of the community is metabolically-activated by rutin. Indeed, we were able to identify a percentage of 7.3 and 26.9% at time 6 and 24 h, respectively, of active cells with respect to the control without any amendment. Translational activity was likely still possible by these cells in the absence of additional amino acids in these short incubations via recycling of internal amino acids (Hatzenpichler et al., 2016).

In our study, the significant bacterial taxa selected are: *Lachnospiraceae* (*Lachnoclostridium*, *Eisenbergiella*), *Entero- bacteriaceae* (*Escherichia*), *Tannerellaceae* (*Parabacte- roides*), and *Erysipelotricaceae* (*Erysipelatoclostridium*). These bacteria may represent a “core rutin-selected microbiota” in healthy individuals. Members of the *Enterobacteriaceae* (*Escherichia coli*, *Escherichia fergusonii*, and *Enterobacter cloacae*) have been previously implicated in the O-deglycosylation and dehydroxylation of different flavonoids (Miyake et al., 1997; Hur et al., 2000; Zhao et al., 2014; Braune and Blaut, 2016). In a batch culture fermentation experiment, Tzounis et al. (2008) found that catechin, a flavan-3-ol monomers, promoted the growth of *E. coli* and Duda-Chodak (2012) found that rutin does not inhibit *E. coli* growth. In accordance with our findings, *P. distasonis* has been shown to produce both Q-glc and Q (Bokkenheuser et al., 1987) and the *Lachnospiraceae* member *Blautia* and *Eubacterium* were able to convert rutin in Q (Kim et al., 2014) and Q-glc in Q, respectively. Based on rutin degradation capability and the identification of the core-rutin selective microbiota, we divided the donor microbiotas into high Q-glc, high Q, and low producers. We observed that members of *Enterobacteriaceae* (*Escherichia*) were associated Q-glc production, putatively due to expression of alpha-rhamnosidases that can act on rutin. We also found that *Lachnospiraceae* (*Lachnoclostridium* and *Eisenbergiella*) were associated with Q production, which may be due to expression of beta-rutinosidase enzymes or a combination of alpha-rhamnosidase and beta-glucosidase enzymes. In agreement with our experimental results, we found that putative alpha-rhamnosidases are common in genomes belonging genera that responded to the rutin amendment.

We did not screen for beta-rutinosidase and beta-glucosidase due to a lack of diagnostic bioinformatic tools for confident general inference of these activities. Beta-rutinosidase has not been identified in bacteria. The closest matches in Bacteria (by BLAST) of confirmed fungal beta-rutinosidases have only weak homology (< 30% identity) and are annotated as cellulases or cellulase-like enzymes. In addition, the carbohydrate-active enzyme (CAZY) database lists nine glycoside hydrolase families with individual members that have been shown to possess beta-glucoside activity (EC 3.2.1.21). However, each of the nine families also contain members that do not have known beta-glucosidase activity. Consequently, it is unclear which of the beta-glucosidases that are listed in CAZY will cleave glucose off of Q-glc specifically.

According to our findings, we hypothesize that inter-individual variability in rutin metabolism is driven by differences in the composition of the gut microbiota. Variation in rutin and other flavonoid metabolisms in humans may also be caused by host and environmental factors as diet and genetic polymorphism and differences in enzymatic activity (Almeida et al., 2018). In conclusion, individual microbiotas exert distinct capability in rutin utilization, showing a higher response in certain individuals, whereas others seem less capable in rutin utilization. Future research that takes into account functional gene analysis, diet, and host physiology will advance our understanding of the role of the gut microbiota in rutin degradation and provide opportunities to improve human health.

## Data Availability Statement

The datasets presented in this study can be found in online repositories. The names of the repository/repositories and accession number(s) can be found below: https://www.ncbi.nlm.nih.gov/, PRJNA622517.

## Ethics Statement

The studies involving human participants were reviewed and approved by the University of Vienna ethics committee (Reference number 00161). The patients/participants provided their written informed consent to participate in this study.

## Author Contributions

DB, LA, and AR conceived and designed the experiments. AR performed the experiments and data analyses. AR and DK performed anaerobic incubation experiments. AS, LW, and AR performed FACS sorting. CH performed bioinformatics analyses. AR and DB wrote the manuscript. All authors gave approval to the final version of the manuscript.

## Conflict of Interest

The authors declare that the research was conducted in the absence of any commercial or financial relationships that could be construed as a potential conflict of interest.

## Footnotes

1 https://www.r-project.org/

2 https://www.uniprot.org

3 http://hmmer.org/

## Abbreviations

FACS: fluorescence activated cell sorting
Q: quercetin
Q-glc: quercetin-3-glucoside.
