# Supplementary material for: Conversion of Rutin, a Prevalent Dietary Flavonol, by the Human Gut Microbiota
Source: Front Microbiol. 2020 Dec 21;11:585428. doi: 10.3389/fmicb.2020.585428 (PMC7779528; doi:10.3389/fmicb.2020.585428)
Supplement: Supplementary file 1 [file Data_Sheet_1.docx]

Supplementary Material


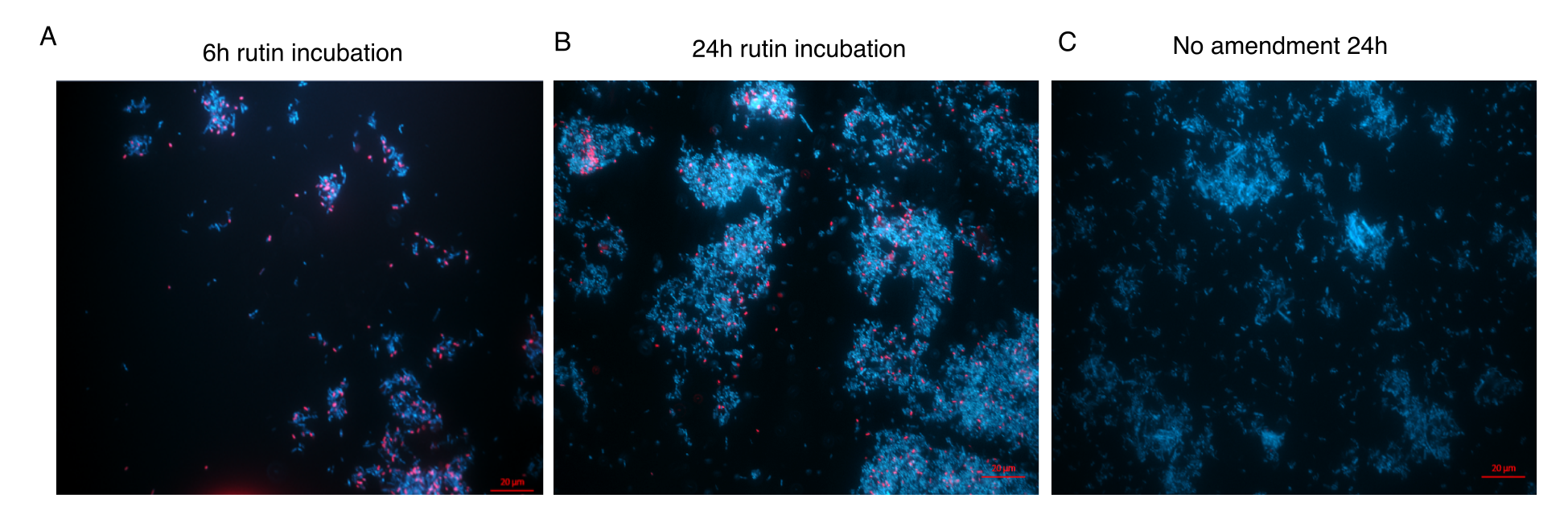


**Supplementary Figure 1.** Representative images of a faecal sample stimulated with rutin. Active cells shows a positive BONCAT signal (pink, Cy5). No amendment control containing dimethyl sulfoxide (DMSO) shows no BONCAT signal. All cells are stained with DAPI (blue).

**Supplementary Figure 2. (A)** Relative abundance (%) of glucose-metabolic active cells in incubations. **(B)** Representative image of a faecal sample stimulated with glucose. Active cells show a positive BONCAT signal (pink, Cy5). All cells are stained with DAPI (blue). Glucose was used as positive controls for each incubation. The percentage of active cells was calculated microscopically (6h: 47.1 ± 7.1%, 24h: 35.6 ± 8.0% [mean ±SD]; t-test: p = 0.0033, n = 20) and relative standard deviation (RSD) across particiants was 0.15 for time 6h and 0.22 for time 24h.


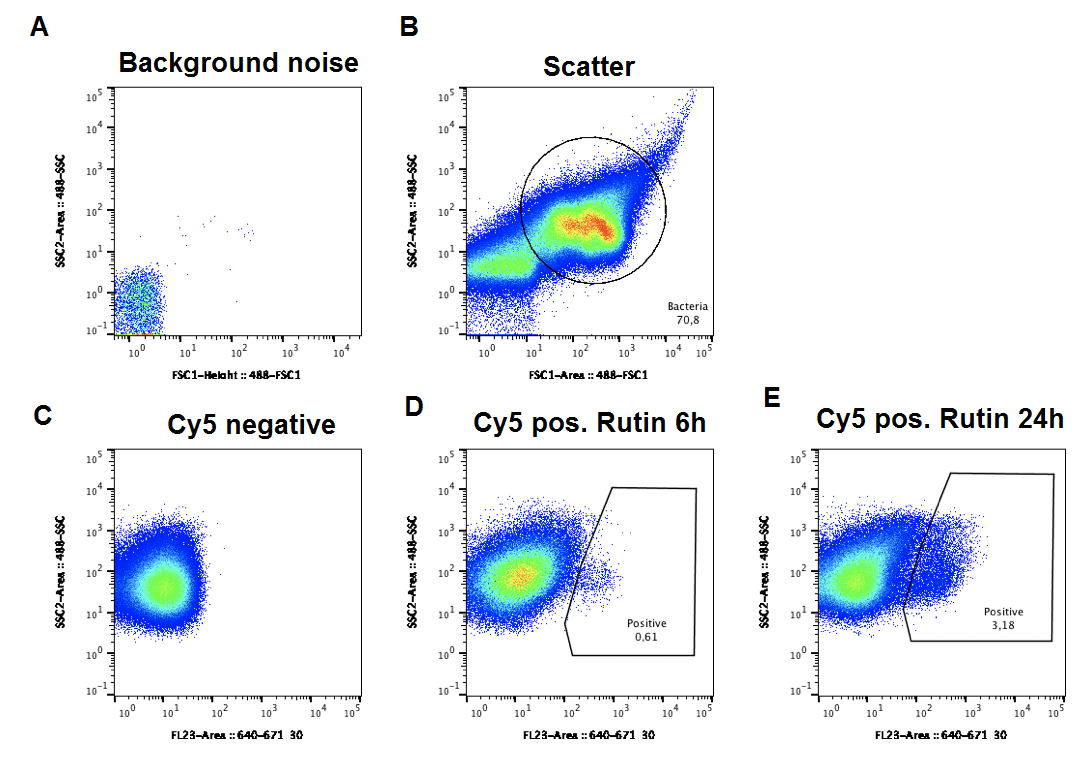


**Supplementary Figure 3**. Bacteria were sorted on a MoFlow Astrios EQ cell sorter. As shown in dotplot (**A**) background noise of the machine was detected using the parameters forward scatter (FSC) and side scatter (SSC). Subsequently bacteria were measured using the same settings and pre-gated as depicted in **(B)**. Then Cy5 negative control bacteria were displayed in a dotplot showing the Cy5 channel via the SSC channel **(C)**. 6h rutin **(D)** and 24h rutin **(E).** Gated Cy5 positive bacteria were sorted.

**
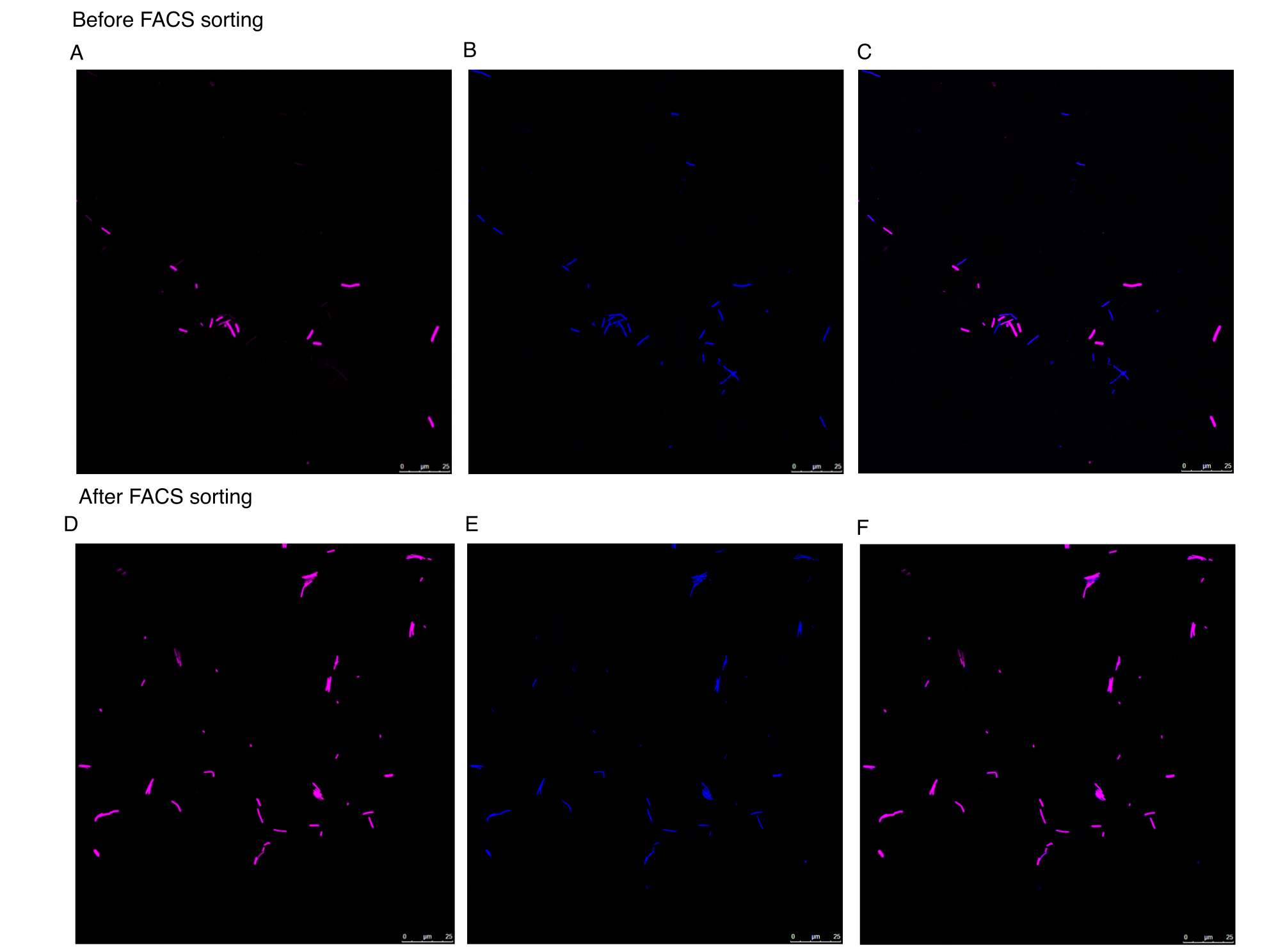
**

**Supplementary Figure 4.** Representative images of a faecal sample stimulated with rutin. Active cells before and after FACS sorting. Before FACS sorting some cells are Cy5 labelled, after FACS sorting all the cells are Cy5 labelled. **(A,D)** Cy5 BONCAT positive (pink), **(B,E)** DAPI-stained cells (blue), **(C,F)** merged images.

**
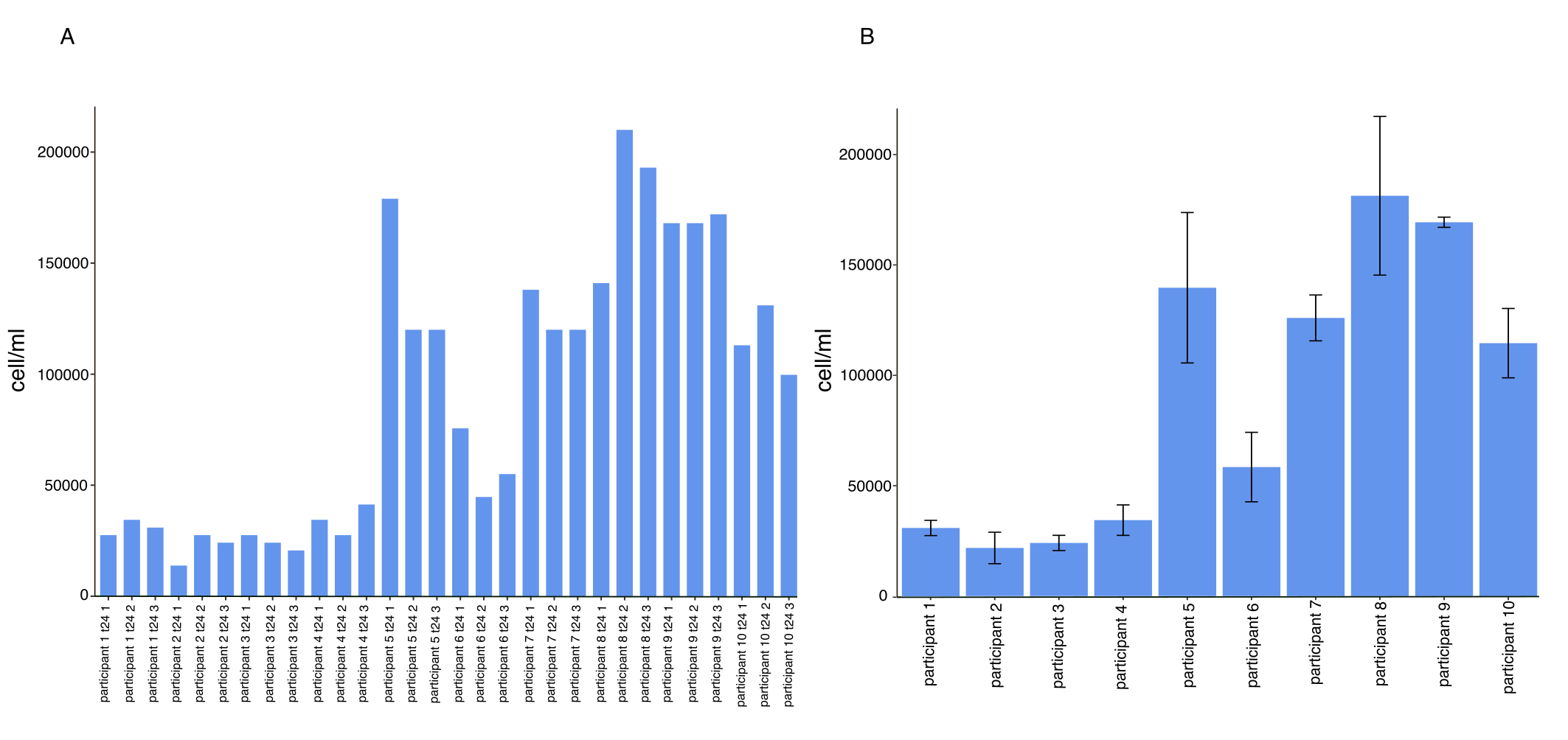
**

**Supplementary Figure 5.** Absolute BONCAT-positive cells count at time 24h performed with FACS. **(A)**The bar-plot shows technical reproducibility of the replicates performed in triplicates for each sample assessing reproducibility of the method. ANOVA: Technical variation: r^2^=0.00085, p=0.878; Biological variation: r2=0.999, p<0.0001. **(B)** The bar-plot shows mean and standard deviation (SD) of the replicates for each participant.

**Supplementary Figure 6.** Prevalence of putative alpha-rhamnosidase. **(A)** Prevalence of putative alpha-rhamnosidase - Bac_rhamnosid6H (PF17389) in publicly available genomes belonging to genera that comprised the core rutin-active community. **(B)** Prevalence of putative alpha-rhamnosidase - Bac_rhamnosid6H (PF17389) in publicly available genomes belonging to genera that include the non-active community. The number of genomes belonging to each genus that was screened is indicated (N=X) and bars indicate the proportion of genomes in which PF17389 could be reliably identified (E<10^-6^ and alignment of at least 300 amino acids). **(C)** Beanplot showing the distribution of alpha-rhamnosidase between the active and the non-active community (active genera: median=0.98, non-active genera: median= 0.53, p=0.041, one-tailed Mann Whitney U test).


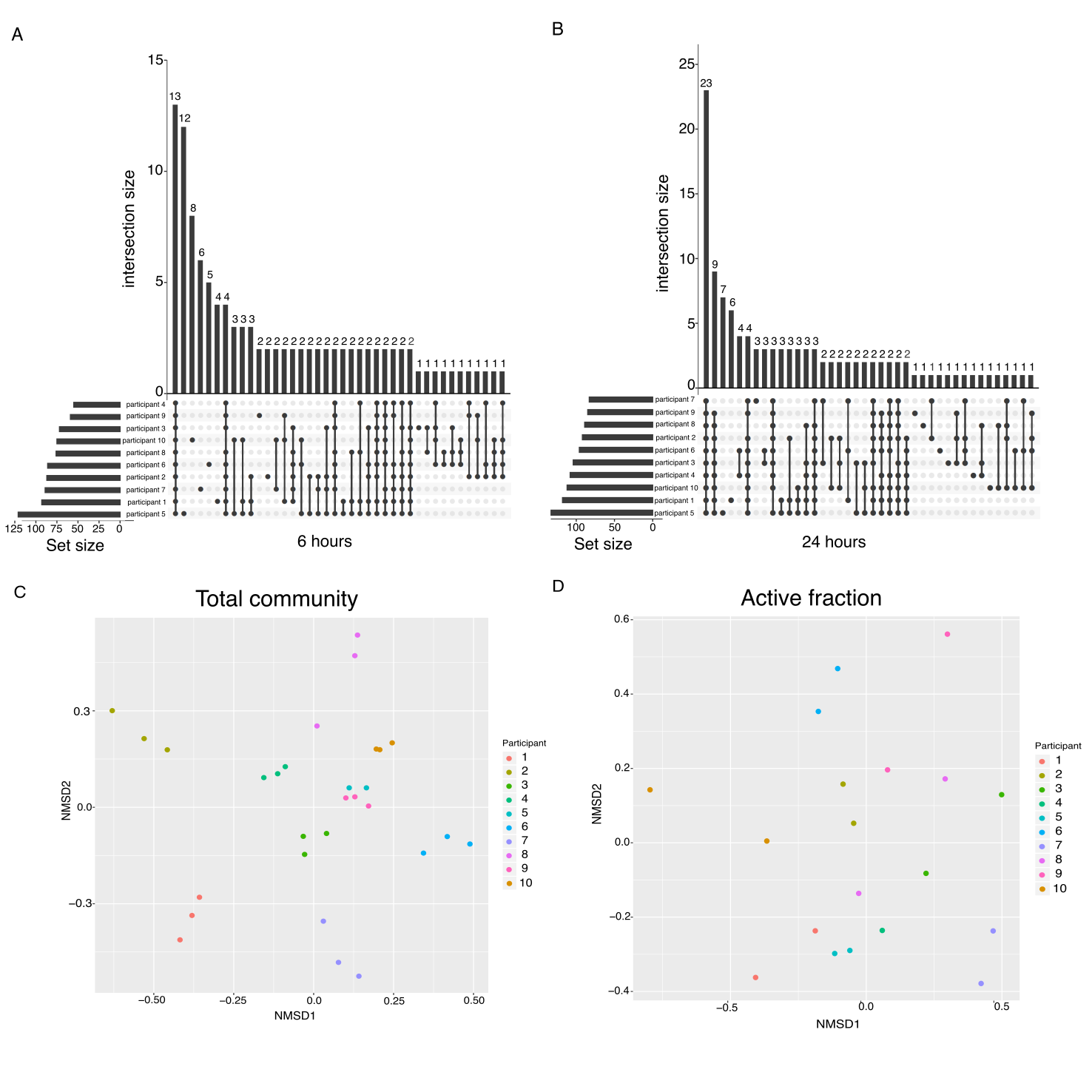


**Supplementary Figure 7.** Individual variability in rutin degradation. **(A)** Active fraction intersections shows 13 ASVs shared between all participants at time 6h and 97 were shared only between some individuals. **(B)** Active fraction intersections shows 23 ASVs shared between all the participant at time 24h and 91 were shared only between some individual. The total size of each set is represented on the left barplot. Every possible intersection is represented by the bottom plot, and their occurrence is shown on the top barplot. Each row corresponds to a set, and each column corresponds to one segment in a Venn diagram. Cells are either empty (light-gray), indicating that this set is not part of that intersection, or filled, showing that the set is participating in the intersection. NMDS ordination shows separation by participant in both total community **(C)** and active fraction **(D)**. The ordination was calculated using Bray-Curtis dissimilarity (perMANOVA, p<0.0001).


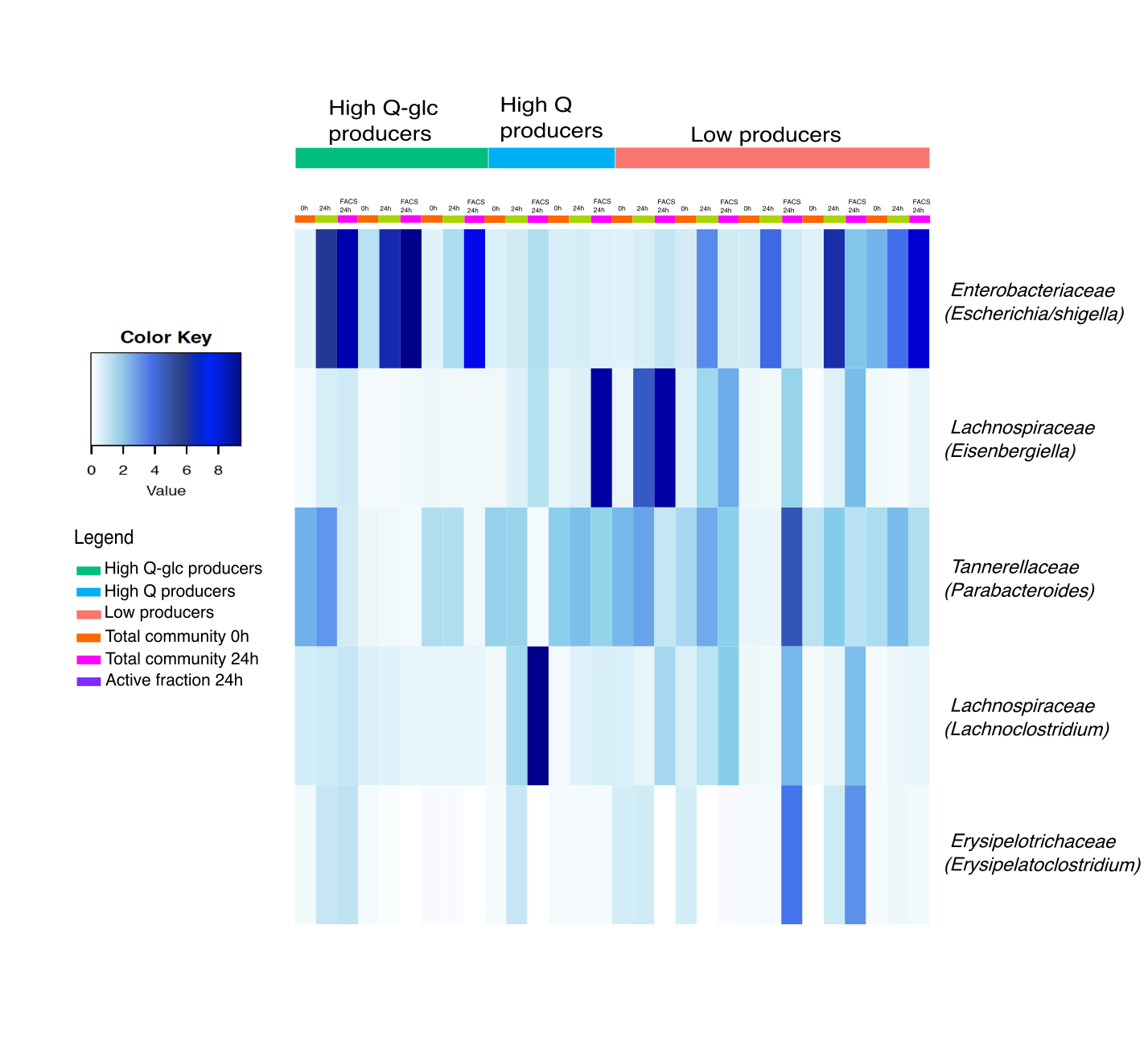


**Supplementary Figure 8.** Genus level heatmap showing the square-root-transformed relative abundance of the active fraction compared to the baseline microbiota at 0h and 24h. Only the genera that were significantly increased in the active fraction compared to the total community are shown (negative binomial distribution, Wald test, p<0.05, ANOVA, p<0.05).
